# “Chipped but not broken” – patients’ symptoms and challenges beyond five years post radiotherapy for meningioma: a qualitative interview study

**DOI:** 10.1186/s12883-026-05049-3

**Published:** 2026-06-16

**Authors:** Linda Åkeflo, Karin Ahlberg, Per Fessé, Petronella Lannerheim, Per Fransson, Ingrid Kristensen, Emma Ohlsson-Nevo, Katarina Sjövall, Ulrica Langegård

**Affiliations:** 1 Institute of Health and Care Sciences, Sahlgrenska Academy, University of Gothenburg, Gothenburg, Sweden; 2 Department of Oncology, Sahlgrenska University Hospital, Gothenburg, Sweden; 3 Centre for Research and Development, Uppsala University/Region Gävleborg, Gävle, Sweden; 4 Department of Haematology, Oncology and Radiation Physics, Lund University Hospital, Lund, Sweden; 5 Department of Nursing, Umeå University, Umeå, Sweden; 6 Department of Clinical Sciences, Oncology and Pathology, Lund University, Lund, Sweden; 7 Department of Surgery, Faculty of Medicine and Health, Örebro University, Örebro, Sweden; 8 University Health Care Research Center, Faculty of Medicine and Health, Örebro University, Örebro, Sweden; 9 Faculty of Health Sciences, Kristianstad University, Kristianstad, Sweden

**Keywords:** Meningioma, Benign brain tumor, Radiotherapy, Proton beam therapy, Qualitative study

## Abstract

**Background:**

Meningioma, the most prevalent brain tumor in adults, is predominantly benign and often treated with radiotherapy and/or surgery, generally with positive treatment outcomes. Radiotherapy can be administered using either conventional radiotherapy (CRT) or proton beam therapy (PBT). The treatment can cause chronic symptoms such as headache, fatigue, and neurocognitive impairments that can significantly affect daily life. However, there is a lack of research on the long-term consequences of treatment, particularly concerning patients’ own experiences of related symptoms. Therefore, this study aimed to explore the experiences and daily life challenges of individuals at five or more years post-radiotherapy for meningioma.

**Methods:**

A qualitative interview study was conducted with 20 participants who were over five years post-treatment and had undergone either PBT or CRT. Using a structured guide with open questions, data were collected through online interviews which were then transcribed verbatim and analyzed using thematic analysis that followed a six-step process described by Braun and Clark.

**Results:**

Of the 20 participants, 17 had received PBT and 3 had undergone CRT, with most having completed their treatment more than six years ago. Two main themes were constructed: “Adjusted life circumstances” and “Reduced autonomy”. Some participants described persistent symptoms such as chronic headaches, cognitive difficulties, and fatigue, which significantly affected their daily lives. Despite these challenges, some patients adapted through establishing new routines, accepting limitations, and altering their perspectives. Reduced autonomy was noted among some, who experienced increased dependence on family, feelings of isolation, and anxiety about tumor recurrence.

**Conclusions:**

Some patients experience persistent symptoms and daily challenges even five or more years after radiotherapy for meningioma. Although some participants adapt by utilizing their own internal and external resources, this can lead to isolation and increased dependence on family and partners. Improving the follow-up care of these patients could help in optimize function and address their challenges.

**Supplementary Information:**

The online version contains supplementary material available at 10.1186/s12883-026-05049-3.

## Background

The treatment for meningioma is commonly associated with positive outcomes in terms of cure and survival; however, it also entails potential side effects [1, 2]. Meningioma, predominantly benign, represents one-third of primary intracranial tumors worldwide and is the most prevalent adult brain tumor [3]. Proton beam therapy (PBT) is an option that is equivalent to conventional radiotherapy (CRT) in terms of effectiveness [4]. Both CRT and PBT carry a risk of side effects, as radiation can affect both the tumor and surrounding tissues. Efforts are continually made to minimize these side effects. CRT uses ionizing radiation, while PBT uses protons, which may allow for more localized energy delivery to the tumor and potentially reduce exposure to healthy tissues [5]. While this suggests a lower risk of side effects, the long-term impacts remain not fully understood.

Annually, approximately 450 individuals in Sweden are diagnosed with meningioma [6]. Asymptomatic cases can be monitored until symptoms occur while the usual treatment modality typically involves surgical resection followed by adjuvant radiotherapy with a dose of approximately 50 Gy. This strategy is especially used in cases of subtotal resection or atypical histology, aimed at reducing the risk of recurrence [2, 7].

The incidence of meningiomas increases with age, with a female predominance for nonmalignant meningiomas [8]. While radiotherapy is lifesaving, it can induce chronic symptoms such as headaches, fatigue, sensory losses, and neurocognitive impairment [1, 9, 10]. In the past decade, research on treatment-induced symptoms has expanded, primarily focusing on short-term effects following treatment. However, there is limited understanding of the long-term symptoms and their impact on patients’ daily lives.

Previous studies of health-related quality of life in meningioma survivors show worse physical functioning compared to healthy controls [11]. Toxicity severity and symptoms are suggested to be linked to treatment rather than tumor type [9, 10]. However, one study reports an increase in symptom severity, with fatigue and depression being the most frequent symptoms three months post radiotherapy [10]. A long-term study (median 38.5 months post-radiotherapy) has shown persistent symptoms, particularly neurological deficits, with a significant proportion of patients experiencing daily functional limitations and social withdrawal. Despite neurological deficits being prevalent, fatigue is reported as the most debilitating symptom in daily life. An initial increase in fatigue has been observed after three months, followed by a significant decrease after 6 months; however, the difference compared to initial levels persisted [6].

Focusing on symptoms beyond five years post-radiotherapy allows for a deeper understanding of their long-term impact on patients’ daily lives. While previous studies have provided valuable insights into outcomes within the first 3 years, they do not fully address the late effects that may persist well beyond this period [9]. With our longitudinal data collection [12], we are uniquely positioned to explore these long-term symptoms in greater depth. This approach aims to bridge a critical knowledge gap, providing clinicians with valuable insights into the prolonged effects of treatment and raising awareness of potential long-term support needs.

While much attention has been given to the negative effects of radiotherapy, it is also worth considering that some patients may experience positive changes in their lives following such challenges. Evans et al. [13] explored post-traumatic growth—personal development following adversity—in patients with various health conditions, including breast, prostate, and colorectal cancer. Their study found that post-traumatic growth was associated with younger age and female gender. However, the concept has yet to be explored in the context of meningioma. Adding this theoretical perspective is suggested to deepen the understanding of typical trauma response [14]. Thus, by considering the potential of post-traumatic growth as one perspective, we intend to broaden the understanding of how patients cope with the effects of radiotherapy.

In an effort to expand our understanding of patients’ long-term experiences, this study aimed to describe how individuals treated with radiotherapy for meningioma experience symptoms and manage the challenges of daily life.

## Materials and methods

### Study design

This study had a qualitative design using thematic analysis to interpret individual interviews [15].

### Participants and procedures

The study participants were selected from the ProtonCare project, a large multicenter research project assessing the role of PBT compared to other modern photon-based radiotherapy techniques. The ultimate purpose of the ProtonCare study group is to investigate patient-reported variables e.g., short-, and long-term symptoms and health-related quality of life in patients receiving PBT [12].

A purposive sampling strategy was used to include individuals with relevant long-term experiences. Of the 31 ProtonCare study participants who had reached at least five years post-radiotherapy at the time of recruitment, all were invited to participate based on their treatment history, resulting in 20 participants being included in the study. Reasons for non-participation (*n* = 11) were health concerns (*n* = 1), feeling exhausted (*n* = 3), hearing impairment (= 1), lack of response to phone calls or text messages (*n* = 5), and unwillingness to discuss the disease (*n* = 1). All patients who agreed to participate provided written informed consent.

### Data collection

An interview guide with open questions concerning participants’ experiences of symptoms and aspects of long-term daily life challenges was designed and utilized. One of the questions explored participants’ current life circumstances in relation to their illness and treatment: “How do you perceive your life today in light of your current illness and previous treatment?” Details of the interview guide are provided as supplementary materials (Additional file 1).

The interviews were conducted by the third author (PL) who has long clinical experience in nursing and radiotherapy; probing questions were also asked to gain more in-depth or detailed answers. Each interview lasted an average of 35 min. All interviews were conducted online via Zoom between 2022 and 2023, with participants utilizing webcams and deciding on the time for the interview. The sample size was regarded as sufficient to address the research question, as data saturation was reached when no novel insights or themes appeared during subsequent interviews. The interviews were audio-recorded and transcribed verbatim by professional transcribers before analysis.

### Data analysis

The transcripts were systematically organized and coded using NVivo 14 software. Thematic analysis as described by Braun and Clark was used to interpret data from individual interviews. An inductive approach was used to explore the experiences of symptoms and distress among individuals with meningioma and to understand aspects of long-term daily life challenges. The analysis followed a six-step process described by Braun and Clark [15, 16]. Step 1: Immediately after each interview, a narrative account of the participant’s story was written by the third author (PL), providing an initial list of ideas and points of interest. The first author (LÅ) then read and re-read the interviews to become familiar with the entire dataset, while searching for patterns and meanings. Step 2: Initial codes were generated iteratively by LÅ by transferring items of interest, questions, connections between data items, and other preliminary ideas reported by the participants to a more conceptual level. Step 3: The coded and collated data extracts were examined for potential themes of broader significance. Themes were developed from the initial codes and discussed with UL. Two of the authors (LÅ, UL) further discussed these codes in relation to patients’ reported experiences. Themes were retained or rejected based on consensus. Quotes were linked to the codes. Step 4: A hierarchical map, including main themes and subthemes, was created (LÅ, UL). Only themes assessed to have adequate commonality were used and coherence was tested for accuracy. Step 5: The mapped themes underwent further review, defining, and refining. After a discussion between the authors offering diverse perspectives, two main themes were established. Narrative description was performed for each theme, including why it was relevant to the broader study question. Step 6: The themes and the underlying story were described in the results, including how the researchers interpreted the data, and supplemented with illustrative quotes. The six-step analysis process was discussed in collaboration with all the authors [11, 12].

## Results

This study aimed to investigate experiences of symptoms and aspects of long-term symptoms, symptom distress, and daily life challenges in patients with meningioma treated with PBT or CRT.

### Characteristics of the study sample

The sample consisted of 14 females and 6 males treated for meningioma with PBT (17 out of 20 participants), while the remaining participants received CRT. For the majority of the participants, more than six years had passed since they completed radiotherapy. Detailed demographic characteristics of the participants are shown in Table 1.

**Table 1:** Demographics of study participants *(n = 20) N= *number of participants

Characteristics		Total study group, *N* = 20
Sex	Female	14
	Male	6
Age	**Mean**: 58.4 years	
	31–40	2
	41–50	2
	51–60	8
	61–70	6
	71–80	2
Treatment modality	Proton	17
	Conventional (photon)	3
Years since completed treatment	**Mean**: 6 years	
	5 years	2
	6 years	9
	7 years	8
	9 years	1
Education level	Elementary school	1
	Secondary school	11
	College/university	8
Employment status	Unemployed	1
	Employed	8
	On sick leave	3
	Disability pension	1
	Retired	7
Marital status	Married or living with a partner	18
	Living alone	2
Children	Living at home	4
	Grown up	8
	No children	4
	Missing	3

### Overview of findings

Two main themes were constructed: (1) Adjusted life circumstances and (2) Reduced autonomy. The results describe the multifaceted nature of their experiences of long-lasting symptoms and symptom distress across various domains of their lives, including the struggles some individuals face due to their loss of autonomy in daily life. The main themes and subthemes are presented in Table 2.

**Table 2:** Themes and subthemes of experiences and daily life challenges at five or more years post radiotherapy for meningioma

Themes	Subthemes
Adjusted life circumstances	Symptoms and symptom distress
	Cognitive and emotional difficulties
	Acceptance
	Shifted perspectives
Reduced autonomy	Roles in relationships and family
	Isolation and social support
	Fear of recurrence

Within the first main theme, *Adjusted life circumstances*, four subthemes were identified: S*ymptoms and symptom distress*,* Cognitive and emotional difficulties*,* Acceptance*, and *Shifted perspectives.* The second theme, *Reduced autonomy*, included three subthemes: *Roles in relationships and family*,* Isolation and social support*, and *Fear of recurrence.*

### Adjusted life circumstances

The first main theme captures the long-term daily life challenges that participants faced during the adaptation process following treatment. These included adjusting to physiological and psychological symptoms, as well as cognitive and emotional difficulties. Additionally, the impact on life perspectives was noted in relation to these adjusted life circumstances.

### Symptoms and symptom distress

Those who had remaining treatment-induced symptoms five years after treatment described chronic headache, impaired vision, sensory loss, fatigue, and reduced energy levels that limited their participation in various activities. The symptoms also emerged as major concerns making it necessary to adapt their daily routines and manage their symptoms. The physical symptoms resulted in limitations in their daily lives and it also seemed important to seek alternative ways to engage in activities.

Yes, it’s the headaches, which can be different. At first, I have this constant chronic pain, so like a pressing pain on one side and then it’s… yes an area as big as a fist maybe, roughly that size. But then on a worse day, for example, then it radiates a lot down behind my eye and down by my ear and in larger areas, and then it can be more cutting and more of a stabbing pain and then the pain can seem to come in several different ways. And that’s when it’s the hardest and that’s when I’m just more or less constantly lying on the couch, so to speak. Participant 2.

…it’s still hard waking up with a headache several days a week, because you’re limited for a few hours in the morning before the tablets start to work. But then I manage it… well, it’s the way it is. I sleep when I get home sometimes because I’m tired. It’s a bit like that… it’s not like I go out in the evening and meet people, I’ve adapted my life, it’s been going on for so many years now that it’s not… I think that, yes, it’s just the way it is, so you have to adapt. Participant 8.

I don’t really have any physical problems as such. But… no, it’s just that you postpone things maybe for a day because no, I don’t have the energy for anything more today, I’ll take it tomorrow. But I don’t see that as a major problem. It’s like I say, you get used to living with the differences, so that… there are always gaps. Participant 17.

### Cognitive and emotional difficulties

Participants reported cognitive and emotional impacts, including memory issues, cognitive impairment, reduced tolerance, and feelings of anger. They described experiencing “brain fatigue”, characterized by cognitive shutdowns and challenges engaging in conversations. A pronounced difficulty in balancing energizing activities with those that drained their energy was also described.

And what I’ve noticed, I mean after the radiotherapy, and I also asked my doctor when I was there last week, I have bad memory problems, it bothers me and the doctors thought that when I started working and, you know, got going, it would get better but it’s like my brain… things don’t stick, so if I’ve done something last week I’ve forgotten that I’ve done it, so it’s like a hole in my head and it bothers me, because it doesn’t matter how much I try to concentrate… I notice that I do learn things but a lot of things become like… it’s like I’ve never done them, it’s never happened. Participant 9.

… then I kind of think that you probably don’t have the same patience or that you can see things like… uh. But you are more… you get angry easily, perhaps or, have a short fuse. Participant 16.

…and my patience and my… that is, my ability to concentrate and so on becomes… it takes a beating over time and in connection with this, I’ve also received a diagnosis of brain fatigue, which is very strong, so it’s like… when it all gets turned off for me, then it goes off, then I can’t manage to think or talk to anyone at all, then it’s over, I just want to be by myself and do nothing or whatever. Participant 2.

### Acceptance

Finding a balance in everyday life and an acceptance of changed energy levels was said to be a dual experience, expressed by several individuals. Participants adopted acceptance as a coping strategy for dealing with their altered circumstances. Recognizing the impact of brain fatigue and tiredness, participants committed to adjusting to their limitations and managing energy levels to maintain a balanced daily life, rather than fighting against them. One participant expressed this as follows:

So I always have to think carefully like, Friday is hardest because then you are tired, but Friday is actually the best day to do something because then you have several days to recover. Participant 4.

But it’s hard to find a balance where it works… I have two children, so you want to get involved in their activities and things like that. So it’s a bit difficult to know whether you should put your energy into work or whether you should put your energy into the family. Well, I think that my energy levels may not be enough to work 100% and be at the office 100% and then also be lively and alert in the evening, I have to take breaks and rest, it’s as simple as that. Participant 8.

Not all study participants coped with the situation by using acceptance as a strategy. As cited below, one participant mentioned adopting a strategy of not discussing the illness, while another expressed feelings about the unfairness of being affected by the disease.

Maybe that’s why I don’t talk about it so much because it makes me so sad. If you don’t talk about it, then it doesn’t exist, that’s a strategy too. Participant 4.

### Shifted perspectives

Participants experienced that a profound re-evaluation of life’s priorities was prompted in the time after completing treatment for meningioma. They felt a shift in perspective, redefining what truly mattered to them.

And then you realize… regardless of a little fatigue and some medicine and a few practical aversions, I know that I’m terribly lucky to have come through this, a little chipped but not broken. It’s also perhaps a reason for, no we’ll stop working now, we’ll enjoy life instead. Participant 3.

And it’s clear that when you’ve got a tumor, you think that life is short and that maybe you get these kinds of thoughts more, if you mean how have I changed my life then it’s even more important that I take care of myself and do things that give me energy and the like, not just keep on going. Life is short, isn’t it? So it’s probably a lesson I’ve learned from everything. Participant 7.

### Reduced autonomy

The second main theme was constructed from participants who reported that they had developed strategies for managing changes in their independence post-treatment. By adapting to newfound limitations, participants appeared to adjust their self-image and autonomy.

### Roles in relationships and family

Participants described feeling increasingly dependent on their partner due to the support needed post treatment. This reliance altered their sense of independence and self-sufficiency. Participants also described observing how their partners compensated for their disabilities.

I need my husband with me to support and push and help me, as I said, he is the one who does most of the work, as you say…. … So of course I’m very dependent on him, I can’t go shopping often and carry heavy bags and rearrange the furniture at home or paint a wall or anything like that, things that I used to be able to do. Participant 2.

… the children have also had to adapt, they were five and seven when I became sick and they’ve never had… it’s often been like this, now mom has a headache, now you have to be quiet, you have to go out, you can’t bring friends round, you know they’ve lived with this for almost as long as they can remember. I have a headache and have to go and rest and that they have to be quiet, the TV can’t be on and it’s still like that, you know, when they’re on their mobile phones and playing a lot of noise and music and stuff like that, they have to turn them off, I have to tell them every day because I don’t want that in the background … So it’s clear, they have to adapt around me as well. Participant 8.

### Isolation and social support

However, participants acknowledged the value of support from healthcare professionals (including psychosocial support and counseling), patient networks, family members, and partners during the challenging times post-treatment.

. But then it was tough when you had to get back 100% (at work ed. note), I thought I would never make it, but I did. But I’ve had to pay for it… because I don’t have the same energy, so I have to isolate myself. Participant 4.

… everyday life with the children… it was so hard to manage everything so then I got some type of counselling. Participant 8.

### Fear of recurrence

Participants expressed fear of tumor recurrence or progression, which could result in reduced autonomy. Moreover, some participants revealed that they had been informed about possible impaired vision in the future due to tumor progression, which would possibly lead to reduced daily functioning and increased reliance on others.

… so of course you can be nervous when you have this …, before you get the results of the MRI. And they’ve said that if it does grow again, then there will be surgery, and then they will take away the sight of that eye, …, … So I know that if it does grow, it grows very slowly in that case, if it grows…, … I will not die, but then my vision in that eye will disappear. Participant 10.

## Discussion

In this qualitative study, we explored the long-term effects of radiotherapy on patients with meningioma, revealing that even five or more years post-treatment, many continue to face persistent symptoms and daily life challenges. While some participants adapted to these circumstances, their reliance on family and partner support remained significant. Previous research on malignant brain tumors highlights the mental burden of cancer treatments, with symptoms such as anxiety, depression, and cognitive impairment [11, 17]. Our study supports the view that meningioma patients similarly endure substantial long-term symptoms, underscoring the potential benefits of individualized follow-up care to improve outcomes.

The main theme, *Adjusted life circumstances*, reflects the necessity for patients to adapt to ongoing challenges after radiotherapy, often requiring a re-evaluation of expectations. *Symptoms and symptom distress*, even six years after treatment, were consistent with those reported in earlier studies within the first three years post-treatment [18]. This observation is further supported by our unpublished longitudinal patient-reported data, which compares baseline data (pre radiotherapy) with data at 60-months post-radiotherapy, revealing slightly increased long-term fatigue and sleep disturbances, alongside marginal improvements in symptoms such as headaches. As highlighted by Frances et al. [17], this underscores the importance of healthcare professionals to recognize the long-term impact of these symptoms. Langegård et al. [19] observed in the immediate post-treatment period, disruptions in family dynamics can occur. In our study, we found that these disruptions are likely to continue to affect participants. Despite these challenges, some participants showed resilience by adopting coping strategies to manage fatigue and balance work and family commitments. For example, participants strategically planned activities to ensure adequate rest and described balancing their energy between work and family commitments.

Cognitive and emotional challenges appeared integral to *Adjusted life circumstances*, influencing participants’ ability to adapt and create a sense of normalcy. Despite memory deficits, concentration issues, and frustration, many participants found ways to balance energizing and demanding activities. Over time, this adjustment likely reduced their concern over their condition. Literature outlines that psychological support can significantly aid patients facing cognitive and emotional challenges [19]. We consider psychological support and participation in support groups could benefit these patients. Additionally, we acknowledge that the current National Clinical Cancer Care Guidelines [20], do not include specific recommendations for meningioma patients, which highlights an important gap in addressing their unique needs. We believe that patients with meningioma may benefit from more structured, specialized follow-up care. Further exploration could provide useful insights into supporting their long-term well-being.

Our findings, over a longer period, align with Langegård et al.‘s work, showing that *acceptance* strategies and inner resilience help participants manage symptoms like brain fatigue, reflecting an evolving approach to coping [21]. Additionally, we found that participants’ social networks are pivotal in supporting patients in managing their energy and daily life balance. This highlights the dynamic interplay between personal resilience and external support in navigating the challenges posed by meningioma over time. Hence, it becomes apparent that participants draw upon a combination of inner resilience and outer strategies to navigate their experiences effectively.

Theoretical perspectives on resilience suggest that coping strategies can lead to post-traumatic growth and reduced distress [22, 23]. However, resilience’s relationship with long-term distress is complex, as reported in a previous study of breast cancer survivors [24]. Our study supports the idea that resilience and adaptive coping positively influence quality of life despite ongoing symptoms after benign brain tumors [19]. Consistent with more recent findings in lower-grade gliomas [25], adjusting, coping, acceptance, and seeking support from family and friends may be used as self-management strategies. Our results align with this, highlighting the importance of self-management in improving quality of life despite persistent symptoms. Several participants also expressed gratitude for their survival and recognized positive aspects in their lives, illustrating the development of a positive view through adaptation and effective coping [14].

The sub-theme *Shifted perspectives* highlights how patients can experience a transformative mental shift during illness adaptation, which may reflect internal resources. This aligns with post-traumatic growth processes, which involve positive psychological changes in response to challenges. These changes can include reshaping values and perceptions, ultimately influencing daily life and symptom management [9, 14]. The development of a positive mindset, linked to resilience, potentially shields against stress and mental illness, improving treatment outcomes, as seen in cancer survivors.

Our finding that individuals redefined what truly mattered to them is consistent with findings in cancer and chronic illness populations [26]. Chronic illness can heighten self-awareness, trigger existential reflections on the past, and prompt concerns about the future. In this study, participants expressed a deeper appreciation for life´s simple pleasures, alongside a sense of gratitude for being alive or experiencing milder symptoms. While existential aspects of post-meningioma treatment remain unexplored, one study provides similar perspectives observed in cancer survivors [27].

The second main theme, *Reduced autonomy*, emerges as a central aspect of participants’ experiences, and relates to aspects of quality of life that incorporate psychological and social dimensions. Our findings are consistent with prior research on patients with malignant brain tumors, who often depend on family support due to physical or cognitive limitations [28]. In our study, participants expressed a reliance on partners or family members. This shift affects roles within relationships and families, prompting adjustments by family members to meet the patient’s needs. There is a possibility that healthcare professionals may not fully recognize the extent of the need for psychosocial support among both patients and their families. Although participants did not express any lack of support, given the *changed roles in relationships and family* reported, these changing family dynamics prompt consideration of risks for patients and their families. Informal caregivers have been studied in malignant brain tumor research, often reporting subjective burdens such as anxiety and new responsibilities [29] while also highlighting the essential role informal caregivers play in supporting patients autonomy [28]. Since the reliance of patients on family support emerged in our study, it is possible that this reliance can impose emotional and physical burdens, raising questions about support network adequacy. While our study did not directly explore caregiver experiences, it is essential to recognize their potential significance. There has been limited research on informal caregivers of patients with meningioma. However, existing findings suggest one third of caregivers’ experience burdens that impact their quality of life, often leading to increased anxiety and depression [29]. Therefore, balancing caregiver support is important to preserve the patient’s independence while also mitigating potential health risks for caregivers.

In the present study, participants expressed *Fear of tumor recurrence*, a sentiment also highlighted by cancer survivors in a study conducted by Heathcote et al. [30]. They likened this fear to living near a potentially eruptive volcano, managing it by recognizing warning signs, akin to “smoke”, while avoiding fixation to preserve overall wellbeing. Considering the potential risk of diminished vision expressed by the study participants, and its implications for autonomy, we should address the complex reality of living with such fear.

### Strengths and limitations

This study has strengths in its focus on participants’ experiences and the use of a methodology well-suited to identifying issues that truly matter to individuals. Conducting online interviews allowed us to include participants regardless of geographical limitations. By conducting interviews in environments familiar to the participants, they were able to respond from the comfort of their own environment, which probably reduced fatigue and overstimulation, resulting in richer and more detailed responses. Since study participants were recruited from a longitudinal cohort study involving patient-reported questionnaires, their heightened symptom awareness is likely to have enriched the interview content with detailed insights. However, there is also the potential for “study fatigue,” where participants may become exhausted by their involvement. This was indicated by three individuals who declined participation.

The study’s limitations include the possibility that symptoms such as cognitive impairment, brain fatigue, and impaired vision may not solely result from radiotherapy, as some of the study participants also underwent surgery. This restricts our ability to isolate radiotherapy effects. Nevertheless, the aim of the study was not to investigate treatment modalities and their impact on patients, but rather to explore the experiences of symptoms, how the participants managed their symptoms, and aspects of long-term daily life challenges. In this regard, the study has contributed by shedding light on the experiences of individuals facing these challenges.

### Clinical implications

The presence of long-term treatment-induced symptoms in meningioma patients presents a significant healthcare challenge. Given that not all patients have access to strong familial support networks, and that reliance solely on informal caregivers may have negative consequences, it is crucial for healthcare professionals to offer dedicated support services and specialized rehabilitation programs. These programs should focus on empowering patients to take control of their situation and strengthening their ability to self-manage, with the ultimate goal of restoring autonomy and improving daily functioning. Areas of focus should include cognitive rehabilitation, pain management, emotional support through psychological counseling, and physical therapy to address mobility or sensory impairments. While some patients demonstrate resilience and post-traumatic growth, the ongoing impact of symptoms on daily life remains significant. We suggest that the development of specialized rehabilitation programs specifically for patients with meningioma, as part of a broader approach to cancer care, could be beneficial, as supported by previous research [10, 11, 29]. As part of such programs, healthcare providers may play a role in identifying informal caregiver burden, offering resources, and providing guidance to support both patients and their families. Further research is needed to better understand the burden on informal caregivers in this context.

### Conclusions

The present study has gone some way towards enhancing the understanding of long-term experiences among individuals treated with PBT or CRT for meningioma. Although the picture remains incomplete, these findings can generate hypotheses for future intervention studies. We suggest that patients may benefit from specialized rehabilitation interventions to optimize function and mitigate treatment-induced late effects. This approach should include the involvement of nurses and psychologists, benefiting both patients and their families. Daily life challenges involve a complex interplay of actions, thoughts, emotions, and adaptation processes. Our exploration reveals that patients continue to rely on their internal and external resources to manage the long-term challenges following meningioma radiotherapy.

## Supplementary Information

Additional file 1.

## Data Availability

The datasets generated and analyzed during the present study are available from the corresponding author on reasonable request.

## Abbreviations

CRT: Conventional radiotherapy
N: Number
PBT: Proton beam therapy
