# Supplementary material for: “Chipped but not broken” – patients’ symptoms and challenges beyond five years post radiotherapy for meningioma: a qualitative interview study
Source: BMC Neurol. 2026 Jun 16;26:391. doi: 10.1186/s12883-026-05049-3 (PMC13270910; doi:10.1186/s12883-026-05049-3)
Supplement: Supplementary file 1 — Additional file 1. [file 12883_2026_5049_MOESM1_ESM.docx]

**Interview Guide for Qualitative Study: ProtonCare-Meningioma**

1. Has your illness and treatment affected your life? If so, how?
2. Are you experiencing any daily challenges related to the illness and previous treatment?
3. Are you experiencing changes in your daily life that may be related to your illness and previous treatment?
4. Has your symptom distress changed over time?
5. How do you manage your symptom distress?
6. Have you experienced any changes in your independence or overall life situation that might be related to your illness and previous treatment?
